# Calcium Influx Inhibition is Involved in the Hypotensive and Vasorelaxant Effects Induced by Yangambin

**DOI:** 10.3390/molecules19056863

**Published:** 2014-05-23

**Authors:** Islania Giselia Albuquerque Araújo, Darizy Flávia Silva, Maria do Carmo de Alustau, Katy Lísias Gondim Dias, Karla Veruska Marques Cavalcante, Robson Cavalcante Veras, José Maria Barbosa-Filho, Mario dos Anjos Neto, Lusiane Maria Bendhack, Nadja de Azevedo Correia, Isac Almeida de Medeiros

**Affiliations:** 1 Laboratório de Farmacologia Cardiovascular, Centro de Biotecnologia, Universidade Federal da Paraíba, Cidade Universitária, 58051-900 João Pessoa, PB, Brazil; 2 Departamento de Ciências Farmacêutica, Centro de Ciências da Saúde, Universidade Federal da Paraíba, Cidade Universitária, 58051-900 João Pessoa, PB, Brazil; 3 Laboratório de Neurociências, Instituto de Ciências da Saúde, Universidade Federal da Bahia, Avenida Reitor Miguel Calmon, Vale do Canela, 40110-902 Salvador, BA, Brazil; 4 Departamento de Fisiologia e Patologia, Centro de Ciências da Saúde, Universidade Federal da Paraíba, Cidade Universitária, 58051-900 João Pessoa, PB, Brazil; 5 Departamento de Farmacologia, Escola de Medicina de Ribeirão Preto, Universidade de São Paulo, postal code Ribeirão Preto, SP, Brazil

**Keywords:** lignan, yangambin, mesenteric artery, vasodilation, calcium influx

## Abstract

The pharmacological effects on the cardiovascular system of yangambin, a lignan isolated from *Ocotea duckei* Vattimo (Lauraceae), were studied in rats using combined functional and biochemical approaches. In non-anaesthetized rats, yangambin (1, 5, 10, 20, 30 mg/kg, i.v.) induced hypotension (−3.5 ± 0.2; −7.1 ± 0.8; −8.9 ± 1.3; −14 ± 2.3, −25.5% ± 2.6%, respectively) accompanied by tachycardia (5.9 ± 0.5; 5.9 ± 1.6; 8.8 ± 1.4; 11.6, 18.8% ± 3.4%, respectively). In isolated rat atria, yangambin (0.1 µM–1 mM) had very slight negative inotropic (Emax = 35.6% ± 6.4%) and chronotropic effects (Emax = 10.2% ± 2.9%). In endothelium-intact rat mesenteric artery, yangambin (0.1 µM–1 mM) induced concentration-dependent relaxation (pD_2_ = 4.5 ± 0.06) of contractions induced by phenylephrine and this effect was not affected by removal of the endothelium. Interestingly, like nifedipine, the relaxant effect induced by yangambin was more potent on the contractile response induced by KCl 80 mM (pD_2_ = 4.8 ± 0.05) when compared to that induced by phenylephrine. Furthermore, yangambin inhibited CaCl_2_-induced contractions in a concentration-dependent manner. This lignan also induced relaxation (pD_2_ = 4.0 ± 0.04) of isolated arteries pre-contracted with S(−)-Bay K 8644. In fura-2/AM-loaded myocytes of rat mesenteric arteries, yangambin inhibited the Ca^2+^ signal evoked by KCl 60 mM. In conclusion, these results suggest that the hypotensive effect of yangambin is probably due to a peripheral vasodilatation that involves, at least, the inhibition the Ca^2+^ influx through voltage-gated Ca^2+^ channels.

## 1. Introduction

**Figure 1:**
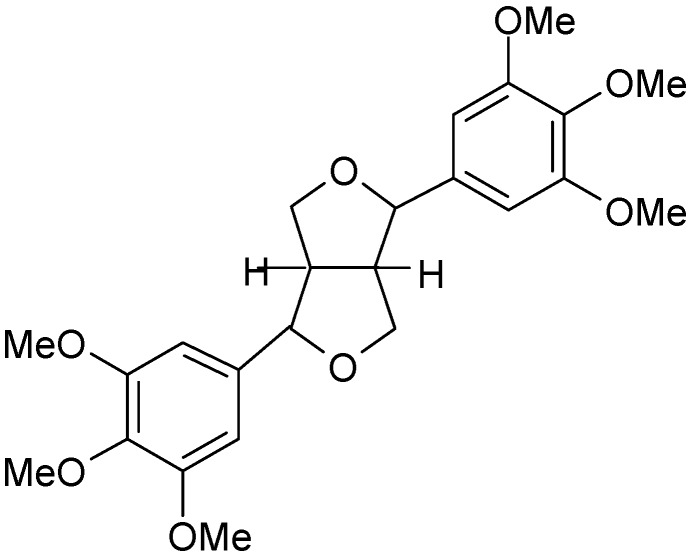
Chemical structure of yangambin.

Lignans are natural products characterized by oxidatively coupled products of two or more phenylpropanoids and can be distinguished by their structural complexity [1]. They can be isolated from all parts of higher plants and are widely distributed in the plant kingdom. Several subgroups are recognized among the lignans, one of the most common is the furofurans such as yangambin (Figure 1) [1]. It is well evidenced that lignans are compounds that show a great potential therapeutic effect on the cardiovascular system [1,2]. It has been reported in the literature that lignans are mainly related to their ability to inhibit cyclic AMP phosphodiesterase [1], activate of endothelial nitric oxide synthase [3], antagonize platelet activating factor (PAF) receptors, block L-type Ca^2+^ channels [1,2] and antioxidant activity [4]. 

Yangambin, a furofuran lignan, was already isolated from plants such as member of the Annonaceae family, including species of the genus Rollinia: *R. pickeli*, *R. exalbida* [5] and *R. mucosa* [6], as well from the *Magnolia biondii*, from the phytogenetically very close family Magnoliaceae [5]. In our laboratory this compound was isolated from the leaves and stem bark of *Ocotea duckei* Vattimo [7]. *Ocotea duckei* Vattimo is a plant of the Lauraceae family found in the northeast of Brazil, and popularly known as “louro de cheiro”, “louro pimenta” and “louro canela”. Traditional medicinal uses of this plant are not known, but several pharmacological studies have shown many properties of yangambin, such as: (a) selective PAF receptor antagonist, observed in several *in vivo* and *in vitro* experimental models [8,9]; (b) protective effects on cardiovascular collapse [10] and anaphylactic shock [11]; (c) anti-allergic effect [12]; (d) depressant effect in the central nervous system [13]; (e) antileishmanial activity [14]. The acute toxicity of the lignan was studied in rodents by Tibiriçá [2]. In mice by either oral or intraperitoneal routes, yangambin at doses up to 1 g/kg, did not induce death within 48 h. However, there is no information in the literature concerning the direct hypotensive or vasodilator activities of this lignan. In this study we examined the hypotensive and vasorelaxant effects of yangambin in rats by using combined functional and biochemical approaches. 

## 2. Results and Discussion

### 2.1. Effect of Yangambin on Blood Pressure

**Figure 2:**
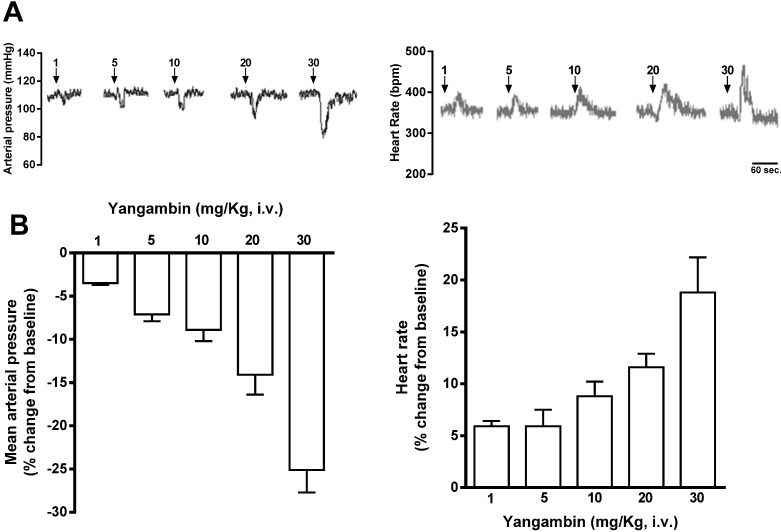
Hypotensive response to yangambin in non-anesthetized rats. (**A**) Representative tracing showing the effects of yangambin on mean arterial pressure and heart rate. The arrows indicate the point at which the doses of yangambin (1, 5, 10, 20, 30 mg/kg) were administered (i.v.). (**B**) Bar graph showing the changes in mean arterial pressure, and heart rate induced by the acute administration of yangambin (1, 5, 10, 20, 30 mg/kg bodyweight, randomly) in non-anesthetized rats. Values are mean ± S.E.M. (*n* = 5).

The effects of yangambin on cardiovascular parameters were studied in non-anesthetized rats. The baseline values of mean arterial pressure and heart rate before yangambin administration were 109 ± 3 mmHg and 363 ± 10 bpm, respectively. Figure 2A and B shows the dose-dependent effect induced by systemic administration of yangambin on mean arterial pressure and heart rate. Yangambin (1; 5; 10; 20, 30 mg/kg i.v., randomly) induced short-lasting hypotension (−3.5 ± 0.2; −7.1 ± 0.8; −8.9 ± 1.3; −14 ± 2.3, –25.5% ± 2.6%, respectively) accompanied by tachycardia (5.9 ± 0.5; 5.9 ± 1.6; 8.8 ± 1.4; 11.6, 18.8% ± 3.4%, respectively). 

### 2.2. Effect of Yangambin on Isolated Rat Atria

**Figure 3:**
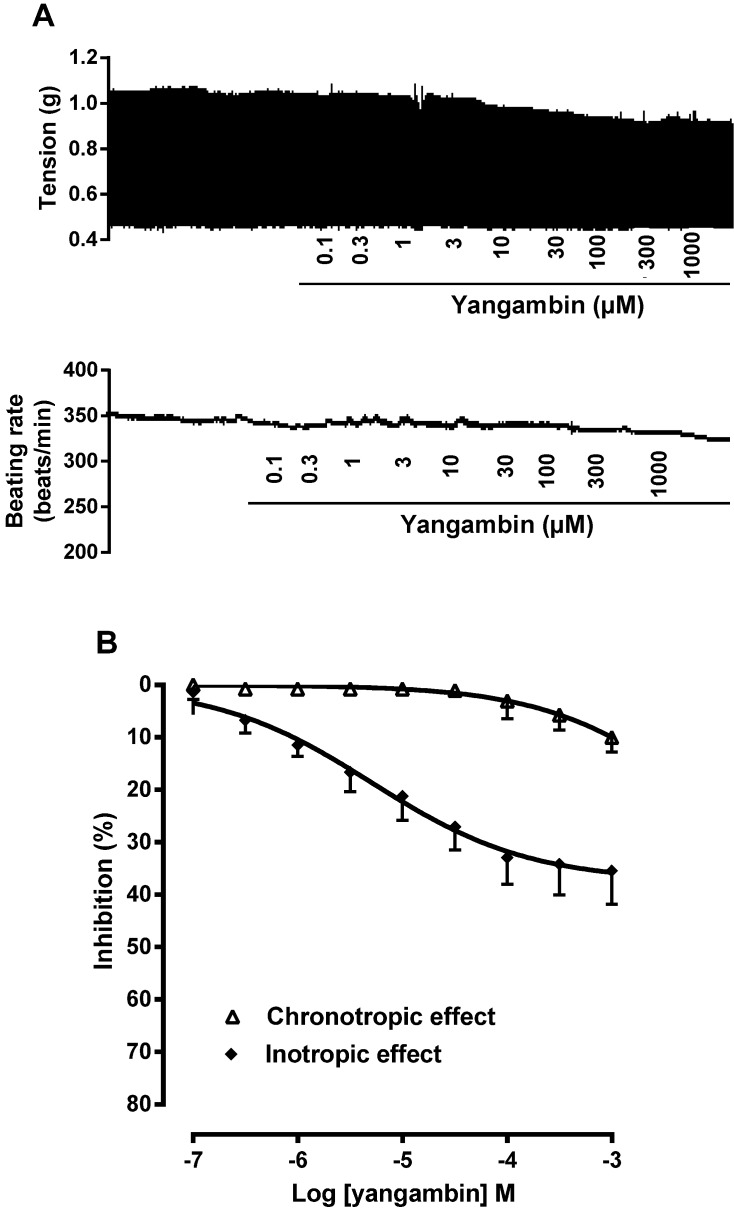
Effect of yangambin on isolated rat atria. (A) Representative tracings showing the effects of yangambin (0.1 µM–1 mM) on left atrial force contraction and right atrial beating rate. (B) Line plot graph showing the negative chronotropic and inotropic effects of yangambin (0.1 µM–1 mM) on isolated right and left rat atria, respectively. Values are mean ± S.E.M. (*n* = 5).

Since the lignan induced a marked blood pressure decrease followed by tachycardia, we hypothesized that increase in heart rate could be reflexive in origin. To investigate a direct cardiac action of yangambin, we designed experiments on isolated rat atrial preparations. In these preparations, yangambin (0.1 µM–1 mM) had very slight negative inotropic and chronotropic effects (Figure 3). The maximal reduction of force and beating rate were about 35.6% ± 6.4% and 10.2% ± 2.9% of the control, respectively). These data reinforce the hypothesis that yangambin-induced tachycardia observed *in vivo* is rather reflex-mediated in response to decrease in blood pressure.

### 2.3. Effect of Yangambin on Rat Superior Mesenteric Arteries

Bearing in mind that a reduction of the peripheral vascular resistance can cause a decrease of the arterial pressure, we hypothesize that yangambin could probably act by relaxing the vascular tissue and thus decreasing peripheral vascular resistances and arterial pressure. To strengthen this hypothesis the vasorelaxant effect of yangambin was evaluated in rat superior mesenteric arteries. 

**Figure 4:**
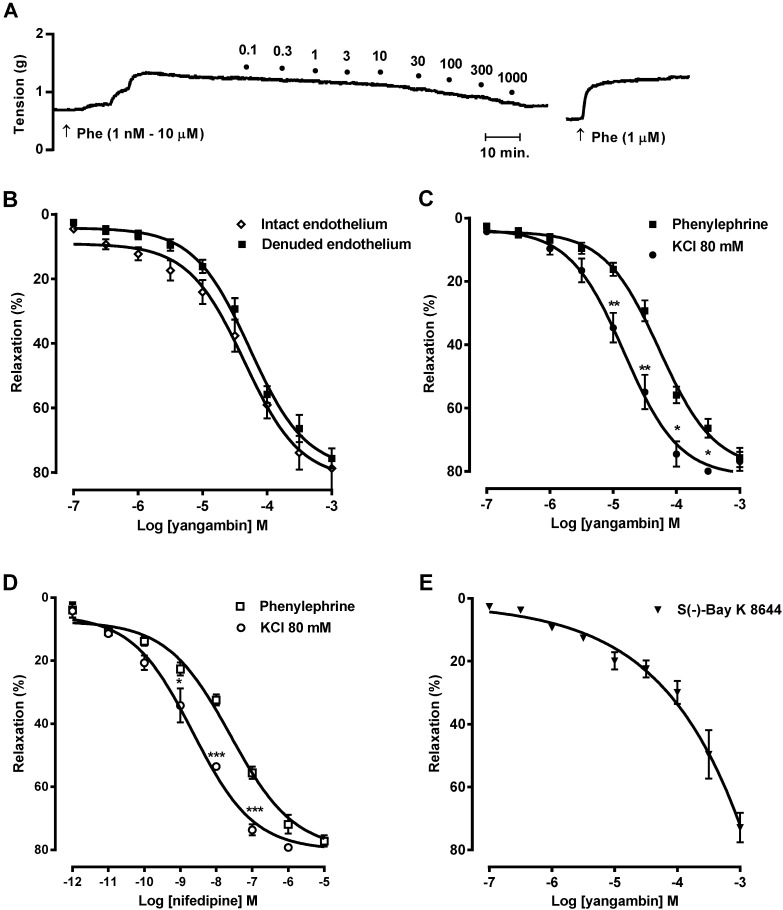
Vasorelaxant effect of yangambin on rat superior mesenteric arteries. (**A**) A typical tracing of the vasorelaxant effect of yangambin on endothelium-denuded arterial ring. Circles indicate the point at which the concentrations of yangambin (µM) were administered. (**B**) Concentration-response curves to yangambin (0.1 µM–1 mM) in rat mesenteric superior artery rings with intact-endothelium (◇, *n* = 9) or denuded-endothelium (▪, *n* = 7), pre-contracted with phenylephrine (1 nM–10 µM). (**C**) Comparison of the vasorelaxing effects of yangambin in endothelium-denuded rings between phenylephrine (1 nM–10 µM) (▪, *n* = 7) or KCl (80 mM) (●, *n* = 7)-induced contractions. (**D**) Comparison of the vasorelaxing effects of nifedipine (1 pM–10 µM) in endothelium-denuded rings between phenylephrine (1 nM–10 µM) (□, *n* = 7) or KCl (80 mM) (〇, *n* = 7)-induced contractions. (**E**) Vasorelaxant effect of yangambin in mesenteric arteries pre-contracted with the voltage-gated Ca^2+^ channels activator S(−)-Bay K 8644 (200 nM) (▼, *n* = 7). Results are expressed as mean ± S.E.M. * *p* < 0.05 *vs.* phenylephrine, ** *p* < 0.01 *vs.* phenylephrine.

In phenylephrine (1 nM–10 µM)-pre-contracted mesenteric rings, yangambin (0.1 µM–1 mM) induced concentration-dependent vasorelaxation (Figure 4). The contractile responses to phenylephrine were reversible after (30 min) washing the rings with Tyrode’s solution (Figure 4A). As shown in Figure 4B, endothelium removal did not alter the vasorelaxing effect of yangambin as demonstrated by the pD_2_ values obtained in arteries with intact endothelium (pD_2_ = 4.5 ± 0.06, *n* = 9) and endothelium-denuded arteries (pD_2_ = 4.4 ± 0.04, *n* = 7). Furthermore, the maximum effect induced by yangambin was not altered after endothelium removal (78.7% ± 2.9% or 75.6% ± 3.1%), indicating that yangambin-induced vasorelaxation may be due to its direct action on the arterial smooth muscle. Considering a lack of influence of the endothelium, all subsequent experiments were performed in vessels denuded of the endothelium.

Interestingly, in endothelium-denuded mesenteric preparations stimulated with 80 mM KCl, the concentration–response curve to yangambin was significantly (*p* < 0.001) leftward shifted (pD_2_ = 4.8 ± 0.05, Emax = 79.9% ± 3.7%) compared with preparations stimulated with phenylepherine (Figure 4C). In view of its stronger inhibitory action on KCl- than on PE-induced contraction, we compared the relaxant effects of yangambin with that of nifedipine, a typical L-type Ca^2+^ channel blocker. Nifedipine (1 pM–10 µM) induced concentration-dependent vasorelaxation in phenylephrine (1 nM–10 µM)-pre-contracted endothelium-denuded mesenteric rings (pD_2_ = 7.8 ± 0.06, Emax = 77.2 ± 2.6, *n* = 9) (Figure 4D). Likewise yangambin, in KCl 80 mM-precontracted endothelium-denuded mesenteric rings, the concentration–response curve to nifedipine was significantly (P<0.001) leftward shifted (pD_2_ = 8.7 ± 0.04, Emax = 80.2 ± 1.6, *n* = 8) compared with preparations stimulated with phenylephrine (Figure 4C). The relaxant effect of yangambin on KCl and phenylephrine-precontracted endothelium-denuded mesenteric rings exhibited a pharmacological profile similar to that produced by nifedipine (Figure 4D). However, nifedipine displayed a more potent effect on inhibition of phenylephrine and KCl-induced contractions than yangambin. Based on this experimental finding, we considered the possibility that yangambin-induced responses could be related to its ability to block voltage-gated Ca^2+^ channels. 

As a matter of fact, lignans block Ca^2+^ channel [1]. In this context, compounds such as syringaresinol mono-β-d-glucoside, extracted from *Boerhaavia diffusa* L. (Nyctaginaceae), which belongs to the same chemical class as yangambin (furofuran lignan), showed significant Ca^2+^ channel blocking effect on frog heart isolated cells, using the whole-cell voltage clamp method [15], 2,3- dibenzylbutane-1,4-diol; a mammalian lignan, inhibited Ca^2+^ channel in rabbit femoral artery [16], schisandrin, the major lignan from *Schisandra chinensis* (Turcz.) Baill. (Schisandraceae) inhibited Ca^2+^ channel in guinea pig ileum [17].

Furthermore, yangambin also induced relaxation of isolated arteries pre-contracted with the voltage-gated Ca^2+^ channels activator S(−)-Bay K 8644 (200 nM) (Figure 4E). The relaxation induced by 1 mM yangambin was 72.9% ± 4.7% (n = 7). These observations provided evidence that yangambin probably blocked Ca^2+^ influx through voltage-gated Ca^2+^ channels in vascular smooth muscle cells, leading to reduction in [Ca^2+^]_i_ in vascular smooth muscle cells.

**Figure 5:**
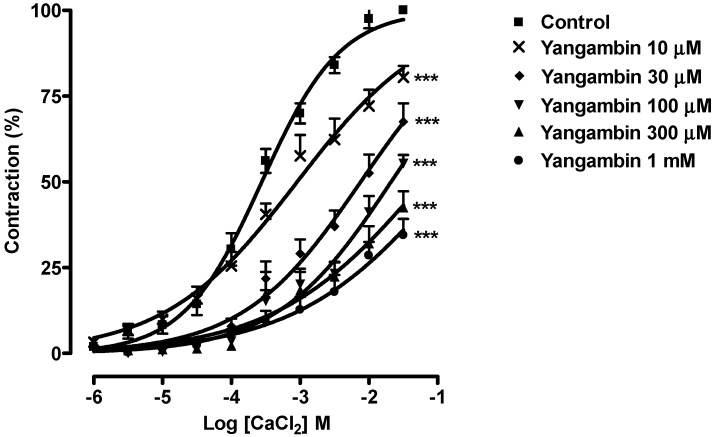
Inhibitory effect of yangambin on CaCl_2_ induced contractile response in endothelium-denuded mesenteric rings. Concentration–response curves for CaCl_2_ were determined in the absence (control) and after the incubation with yangambin at 10, 30, 100, 300 μM or 1 mM (n=5). Results are expressed as mean ± S.E.M. *** *p* < 0.001 *vs.* Control.

In order to provide further evidence for the involvement of Ca^2+^ influx through voltage-gated Ca^2+^ channels in the relaxation induced by yangambin, we examined their effects on contractions induced by cumulative addition of Ca^2+^ to KCl (60 mM)-stimulated vessels. Contractions to Ca^2+^ occur mainly through Ca^2+^ influx following activation of voltage-gated Ca^2+^ channels in the vascular smooth muscle. Figure 5 shows that in depolarizing nominally Ca^2+^-free medium cumulative addition of CaCl_2_(1 µM–30 mM) caused concentration-dependent contractions of mesenteric rings. When the mesenteric rings were pretreated with different concentrations of yangambin (10, 30, 100, 300 μM or 1 mM) the concentration-response curves to CaCl_2_ were attenuated in a concentration-dependent manner, suggesting that Ca^2+^ influx through voltage-gated Ca^2+^ channels was probably inhibited. 

### 2.4. Effect of Yangambin on KCl (60 mM)-Induced [Ca^2+^]_i_ Increases in Fura-2/AM-Loaded Mesenteric Smooth Muscle Cells

**Figure 6:**
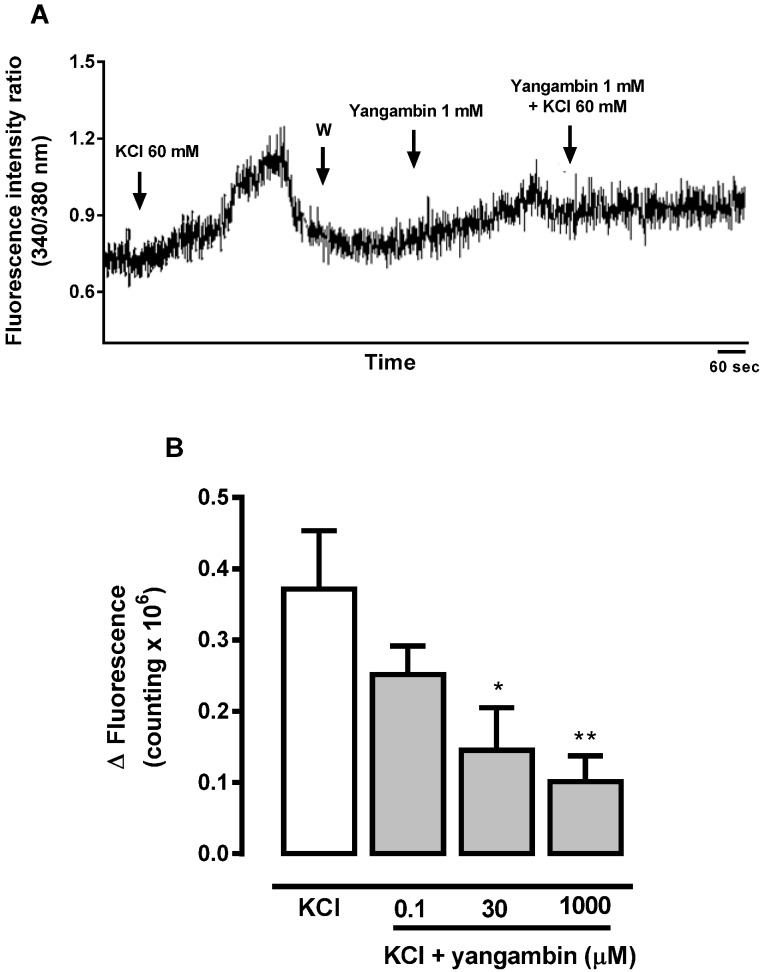
(**A**) Representative tracing showing the inhibitory effect of yangambin on the Ca^2+^ signal evoked by KCl 60 mM in fura-2/AM-loaded mesenteric smooth muscle cell. (**B**) Bar graph showing the inhibitory effect of yangambin on the Ca^2+^ signal evoked by KCl 60 mM. Results are expressed as mean ± S.E.M. (*n* = 3). * *p* < 0.05 *vs.* KCl, ** *p* < 0.01 *vs.* KCl.

Depolarization of fura-2/AM-loaded rat mesenteric vascular smooth muscle cells by high KCl (60 mM) solution simultaneously increased the [Ca^2+^]_i_ (Figure 6).KCl (60 mM)-induced Ca^2+^ signal was significantly reduced by yangambin (0.1 µM, 30 µM, 1 mM) as compared to control in a concentration dependent manner (Figure 6), indicating that the effect of the lignan on blood vessels was probably related to interference with Ca^2+^ influx through voltage-gated Ca^2+^ channels, leading to reduction in [Ca^2+^]_i_.

Interestingly, assuming a total blood volume of about 19 mL in a 300 g rat [18], a peak blood yangambin concentration of 1 mM can be expected at the 30 mg/kg body weigth dose, which corresponds to the maximum value for the vasorelaxant effect induced by the lignan. Taken together, the present findings strongly suggest that the arterial vasodilation, induced by inhibition of Ca^2+^ influx into vascular smooth muscle cells, represents the main mechanism for the hypotensive effect of yangambin. Finally, it is well reported in the literature that yangambin have been mainly related to their ability to antagonize PAF receptors [2,8,9], nevertheless, this is the first sudy to report the inhibition of calcium influx induced by yangambin.

## 3. Experimental

### 3.1. Animals

Male Wistar rats (300-350 g) were used for all experiments. Animals were housed under conditions of controlled temperature (21 ± 1 °C) and lighting (lights on: 06:00–18:00 h). In addition, they had free access to food (PURINA, São Paulo, Brazil) and tap water *ad libitum*. Experimental protocols and procedures were approved by Laboratório de Tecnologia Farmacêutica Animals Care and Use Committee (n 0201/07).

### 3.2. Yangambin

Yangambin (mol. wt. = 446) spectroscopically pure was isolated from the leaves and stem bark of the *Ocotea duckei* Vattimo (Lauraceae) according to the procedures previously described by Morais *et al.* [7], Yangambin was solubilized in a mixture of distilled water/Chremophor (for functional studies) or water/Tween 80 (for biochemical studies) and diluted to the desired concentrations with distilled water just before the use. The final concentration of Chremophor or Tween 80 in the bath never exceeded 0.1% and has been shown without effect when tested in control preparations.

### 3.3. Direct Blood Pressure Measurements in Normotensive Non-Anaesthetized Rats

Intra-aortic blood pressure was recorded by using a technique described by Queiroz *et al.* [19]. Briefly, under sodium thiopental anesthesia (45 mg/kg, i.v.), the lower abdominal aorta and inferior vena cava were canulated via the left femoral artery and vein using polyethylene catheters. Thereafter, catheters were filled with heparinized saline solution and led under the skin to emerge between the *scapulae*. Arterial pressure was measured after 24 h by connecting the arterial catheter to a pre-calibrated pressure transducer (Statham P23 ID; Gould, Cleveland, OH, USA) coupled to an amplifier (Model TBM-4M, WPI, Sarasota, FL, USA.) and connected to a computer equipped with an analog to digital converter board (CIO-AS16/JR, Computer Boards, Inc., Mansfield, MA, USA) and CVMS software (WPI). The data were sampled at a frequency of 500 Hz. For each cardiac cycle, the computer calculated mean arterial pressure (MAP) and heart rate (HR). The venous catheter was used for drug administration. Sodium nitroprusside (10 μg/kg, i.v.) was injected to check the efficacy of the venous catheter insertion. Experiments were performed 24 hafter the surgery.

To evaluate the systemic hemodynamic effects induced by yangambin in conscious non-anesthetized rats, after the cardiovascular parameters had been stabilized, the mean arterial pressure and heart rate were recorded before (baseline values) and after administration of yangambin (1, 5, 10, 20, 30 mg/kg, i.v., randomly). Successive injections were separated by a time interval sufficient to allow full recovery of arterial pressure, usually 15–20 min.

### 3.4. Preparation of Rat Atrial Muscle

Rat atria were isolated and perfused according to the technique described by [20]. Briefly, rats were killed by cervical dislocation and the heart was rapidly excised. The whole left atrium and whole right atrium were cut perpendicular to the axis of the heart, and each atrium was suspended in an organ bath containing Krebs bicarbonate solution. The organ bath was maintained at 37 °C and gassed with a mixture of 95% O_2_ and 5% CO_2_. The resting tension of each atrium was adjusted to 500 mg and the tissues were equilibrated for at least 60 min before the experiments. The left atrium was driven electrically through two parallel platinum electrodes by rectangular pulses with a frequency of 3 Hz, a duration of 3 ms and a voltage of 1.5-fold threshold. The isometric tension was measured and recorded using a force–displacement transducer (Ugo Basile, Comerio, VA, Italy) coupled to a physiograph (Gemini 2, Ugo Basile). The composition (mM L^−1^) of the *Krebs bicarbonate solution* (*KBS*) was as follows: NaCl 118.4; KCl 4.7; NaHCO_3_ 25.00; CaCl_2_·2H_2_O 2.5; glucose 10.0; NaH_2_PO_4_·H_2_O 2.5 and MgSO_4_·H_2_O 1.2. Cumulative concentration–response curves to yangambin (0.1 µM–1 mM) were constructed by stepwise addition of lignan. In some experiments, the rate of spontaneous beating of right atrium was measured, being defined as atrial rate, in order to assess the chronotropic efftects of the lignan. The inotropic effect of yangambin was studied in the electrically stimulated left atrium. Both the chronotropic and inotropic effects were observed 5 min after addition of the compound.

### 3.5. Vascular Tension study in Rat Superior Mesenteric Artery Rings

The superior mesenteric arteries were removed and cleaned from connective tissue and fat. The vessel was cut into ring segments of about 1-2 mm. When necessary the endothelium was removed by gently rubbing of the intimal surface of the vessels with moistened cotton strings. Mesenteric rings (1–2 mm) were obtained and suspended by cotton threads in organ baths containing 10 mL Tyrode’s solution of the following composition (in mM): NaCl 158.3; KCl 4.0; CaCl_2_ 2.0; MgCl_2_ 1.05; NaH_2_PO_4_ 0.42; NaHCO_3_ 10.0 and glucose, 5.6 (pH = 7.4), maintained at 37 °C and gassed with carbogenic mixture (95% O_2_ and 5% CO_2_). Rings were stabilized under a resting tension of 0.75 g for 1 h. During this time the solution was changed every 15 min to prevent the accumulation of metabolites that could otherwise lead to misinterpretations. The isometric contraction was recorded by a force transducer (FORT-10, WPI) coupled to an amplifier-recorder (Transbridge-4, WPI) and to a personal computer equipped with an analog–to–digital converter board. The presence of functional endothelium was assessed by the ability of acetylcholine (10 µM) to induce more than 90% relaxation of vessels pre-contracted with phenylephrine (10 µM) and the absence of relaxation to acetylcholine was taken as an evidence that the vessel segments were functionally denuded of endothelium. 

In the first set of experiments, the effect of yangambin on sustained contractions induced by phenylephrine (1 nM–10 μM) in isolated preparations from rat superior mesenteric arteries was evaluated. After equilibration period, the rings with or without functional endothelium were pre-contracted with the agonist and once the response to the second administration of phenylephrine (1 nM–10 μM) had reached the plateau, increasing cumulative concentrations of yangambin (0.1 µM–1 mM) were added to the bath. The relaxations were measured by comparing the developed tension before and after addition of yangambin.

In the second set of experiments, the vasorelaxant effect of yangambin was also carried out in endothelium-denuded mesenteric rings precontracted with KCl (80 mM) or L-type voltage-gated Ca^2+^ channels activator S(−)-Bay K 8644 (200 nM). The contractions to this Ca^2+^ agonist were obtained in a medium that contained KCl 20 mM [21]. This medium was used because a partial depolarization of the cell membranes is necessary to obtain contractile responses to S(−)-Bay K 8644 [22]. 

The vasorelaxant effects of L-type Ca^2+^ channel blocker, nifedipine (1ρM–10 µM), were also tested on endothelium-denuded mesenteric rings pre-contracted with either phenylephrine 1 nM–10 μM) or KCl (80 mM).

To investigate the effect of yangambin on the contraction dependent on Ca^2+^ influx through voltage-gated Ca^2+^ channels we performed experiments, in endothelium-denuded superior mesenteric rings, under nominally Ca^2+^-free conditions. Extracellular Ca^2+^ was removed by washing vessels with nominally Ca^2+^-free Tyrode's solution (composition the same as Tyrode’s solution, but with CaCl_2_ omitted) and then exposed for an additional 15 min to a high-K^+^ (60 mM) nominally Ca^2+^-free solution. Soon afterwards, the cumulative concentration–response curve for CaCl_2_ (ranging from 1 μM to 30 mM) was then obtained. As concentration-response curves to CaCl_2_ were found to be reproducible in any given vessel, after a control curve and the washing process, a second test curve was constructed in the presence of yangambin (10, 30, 100, 300 μM, 1 mM) (with 15 min pre-incubation). Each preparation was exposed to only one yangambin concentration. The maximal contraction obtained with the control concentration–response curve for CaCl_2_ was taken as 100%, and all values were calculated as a percentage of the maximal response.

### 3.6. Preparation of Mesenteric Smooth Muscle Cells

The vascular smooth muscle cells were isolated from rat superior mesenteric artery were isolated by enzymatic digestion. In brief, the rat superior mesenteric arteries were dissected and longitudinally opened. The endothelium and the adventitia were removed, and the tissue was minced into small pieces, which were incubated in Ca^2+^-free Hank’s solution containing collagenase type II-S (0.03 mg/mL). The tissue was gently shaken in this solution for 20 min at 37 °C and bubbled with a carbogen mixture. After that, bovine serum albumin (type I, 10 mg/mL) was added to the vessel fragments present in Ca^2+^-free Hank’s solution, and the cells were released by mechanical dispersion with a Pasteur pipette. The resultant cell suspension was centrifuged at 1000 rpm for 3 min and suspended in Dulbecco’s modified Eagle’s medium containing glutamine (2 mM), HEPES (pH 7.4, 20 mM), penicillin (10,000 U/mL), and streptomycin (10,000 ug/mL) [23]. The cells were plated on glass coverslips pre-treated with poly-L-lysine 1/10 and kept in a humidified atmosphere (5% CO_2_), at 37 °C, and used 4 h after plating, in a serum-free medium.

### 3.7. Loading of Vascular Smooth Muscle Cells with Fura-2/AM

[Ca^2+^]_i_ was measured with the fluorescent dye Fura-2/AM. The vascular smooth muscle cells were incubated with 5 µM Fura-2/AM for 40 min in a humidified atmosphere (5% CO_2_) at 37 °C. After loading with Fura-2/AM, the cells were immersed in Hank’s solution and placed in recording chambers maintained at 37 °C equipped with stage of a leitzdiavert inverted fluorescence microscope. Fluorescence Fura-2/AM emission was excited alternately at 340-nm and 380-nm bandpass filters mounted in a computer controlled by filter wheel (Lambda-10, Sutter Instruments, Novato, CA, USA). The cells that showed fluorescence above 10^6^ counts were used. At least three independent experiments were performed using separate cells. To study the effect of yangambin on Ca^2+^ influx through voltage-gated Ca^2+^ channels, the vascular smooth muscle cells were stimulated with KCl (60 mM) before and after pre-incubation with yangambin (0.1 μM, 30 μM, 1 mM), and changes in [Ca^2+^]_i_ were recorded. 

### 3.8. Data Analysis

Values are expressed as mean ± S.E.M.. Inotropic and chronotropic effects are expressed as % change of basal contractility of the left atrium and spontaneous beating rate of right atrium, respectively. Student’s t-test or one-way ANOVA were used to compare 2, 3, or more groups, respectively, with the addition of Bonferroni’s multiple comparisons post-test using GraphPad Prism 5.0 (Graph Pad Software, Inc., La Jolla, CA, USA). Two-sided *p* < 0.05 was considered statistically significant. The pD_2_ (-Log EC_50_) value was calculated from the fitted sigmoidal curves.

## 4. Conclusions

In conclusion, the present study, using combined *in vivo* and *in vitro* approaches, demonstrated that yangambin induced hypotensive and tachycardic effects in normotensive non-anaesthetized rats. Hypotension may probably be due to the inhibition of Ca^2+^ influx through voltage-gated Ca^2+^ channels, leading to the reduction in [Ca^2+^]_i_ in vascular smooth muscle cells and consequent peripheral vasodilation.
